# Graphene-Based Kinetic Promotion of Gas Hydrate Formation

**DOI:** 10.3389/fchem.2020.00481

**Published:** 2020-06-19

**Authors:** Meng-Ting Sun, Guo-Dong Zhang, Fei Wang

**Affiliations:** Shandong Engineering Laboratory for Preparation and Application of High-Performance Carbon-Materials, College of Electromechanical Engineering, Qingdao University of Science and Technology, Qingdao, China

**Keywords:** gas hydrate formation, graphene, promoter, kinetics, interfacial transfer

## Abstract

Gas hydrate technology holds great potential in energy and environmental fields, and achieving efficient gas hydrate formation is critical for its industrial application. Graphene is a novel carbon-based nanostructured material with excellent thermal conductivity and a large specific surface area. Therefore, the use of graphene-based materials for the promotion of gas hydrate formation might be feasible and has aroused a lot of interests. Accordingly, to evaluate the current research on graphene-based promotion of gas hydrate formation, this work presents a review of existing studies involving graphene-based promoters of gas hydrate formation. Here, the studies applying various types of graphene-based promoters for gas hydrate formation are listed and detailed, the peculiar properties of graphene-based promoters are discussed, and the promotion mechanisms are analyzed. Through this review, comprehensive insight into graphene-based promotion of gas hydrate formation can be obtained, which can guide the design and applications of novel graphene-based promoters and might contribute to achieving efficient gas hydrate formation.

## Introduction

**Figure 1:**
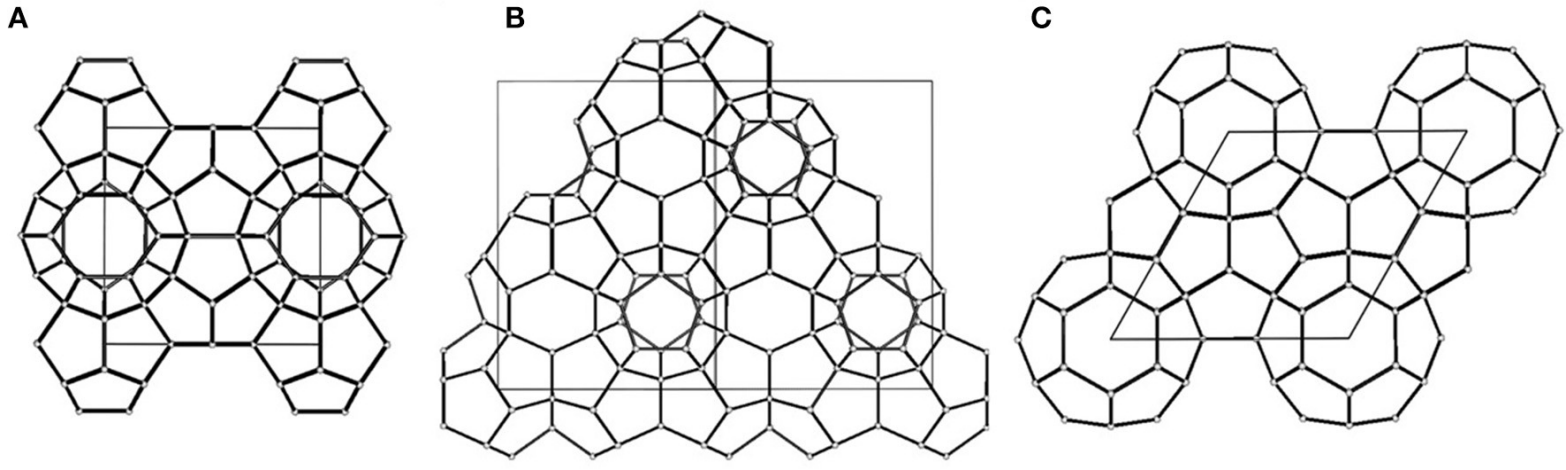
Crystal structure types of gas hydrates: **(A)** Structure I, **(B)** Structure II, **(C)** Structure H; the solid line in each figure represents the unit cell of gas hydrates; reproduced from Takeya et al. (2009) with permission from the American Chemical Society.

Gas hydrates have captured an increasing amount of attention during the past decades because of their great potential for energy storage and environmental conservation (Li et al., 2019). Gas hydrates are ice-like crystalline compounds formed by water molecules (hosts) and gas molecules (guests) under favorable conditions. Water molecules form cage-like vacancies via hydrogen bonds and trap gas molecules in vacancies via Van der Waals forces (Sun et al., 2003). Commonly, based on the crystal structures of hydrates, gas hydrates are considered to have three representative types: structure I, structure II, and structure H (Figure 1; Takeya et al., 2009). Gas hydrates can achieve high storage capacity and are stored under mild conditions and accordingly are considered to be highly promising media for gas separation, gas storage and transportation, and carbon capture and sequestration (Zhong and Rogers, 2000; Li et al., 2019). The gas hydrate formation process (HFP) involves two stages: the nucleation period and the growth period. During the nucleation period, the hydrate crystals are formed by gas and water molecules. However, these hydrate crystals are not stable until they grow to critical sizes, which leads to a stochastic and long nucleation period. After that, rapid hydrate growth is achieved, and a hydrate film is initially formed at the gas–liquid interface, which hinders the diffusion of gas into liquid and consequently results in a slow hydrate formation rate and low water to hydrate conversion. The stochastic induction time and the low formation rate are the main issues impeding the industrial application of gas hydrates (He and Wang, 2018). Therefore, achieving efficient gas hydrate formation is essential for the industrialization of gas hydrate technology.

Because HFP is an interfacial phenomenon, increasing heat or mass transfer in the gas–liquid interface can effectively promote the hydrate formation rate. Mechanical methods, which include stirring, water spraying, and gas bubbling, can achieve rapid hydrate growth via improving mass transfer between gas, and liquid. However, mechanical methods consume energy, which leads to increased cost and, at the same time, generates frictional heat in the system, which works against the exothermic hydrate formation (Fukumoto et al., 2001; Luo et al., 2007; Zhong et al., 2015). During the past two decades, increasing research interest has been paid to promoters, which act as non-mechanical methods to improve HFP. Promoters are divided into two categories: thermodynamic promoters and kinetic promoters (He et al., 2019). Thermodynamic promoters, including tetra-n-butyl ammonium halide (TBAH) and tetrahydrofuran (THF), enhance hydrate formation via reducing the phase equilibrium conditions and moderating the reaction conditions (Joshi et al., 2012; da Silva Lirio et al., 2013). Kinetic promoters improve heat or mass transfer during HFP and consequently speed up the hydrate formation rate (Nashed et al., 2018). Various surfactants have been applied to facilitate the dissolution of gas in water by reducing the mass transfer resistance and have resulted in an improved hydrate formation rate and a reduced induction time. Among these, sodium dodecyl sulfate (SDS) performed best in promoting HFP (He et al., 2019). However, the surfactants generate a large amount of foam in the system and cover the gas–water interface, which reduces the dissolution of gas in water as well as causing losses of surfactants (Veluswamy et al., 2016).

Recently, carbon nanostructures have been demonstrated to be efficient promoters of gas HFP without causing the foaming problem (Park et al., 2010). On the one hand, HFP is exothermic, and the heat generated during the process will detroy the hydrate crystals and negatively impact hydrate growth; therefore, carbon nanostructures with high thermal conductivity can eliminate the heat from the system, which maintains the system at a low temperature and makes the hydrate growth more stable. On the other hand, the carbon nanostructures exhibit a large specific surface area due to their nanometric shape and size, which provides more active sites for nucleation and consequently increases mass transfer. Furthermore, the inhomogeneity of the system will rise in the presence of carbon nanostructures, and heterogeneous nucleation will occur, which forms hydrate crystals more easily than homogenous nucleation. Accordingly, the HFP can be improved by carbon nanostructures (Ghozatloo et al., 2015; Rezaei et al., 2016).

As a novel carbon nanostructure, graphene presents excellent mechanical strength and thermal conductivity and large specific surface area, making it a promising candidate for the promotion of gas hydrate formation (Wang et al., 2017). Here, we implement a review focusing on graphene-based promoters of gas hydrate formation. We initially introduce the exceptional properties of graphene-based materials; we then expound the cases where different graphene-based promoters have been used for gas hydrate formation and discuss their promotion mechanisms in detail.

## Properties of Graphene and Related Materials

**Figure 2:**
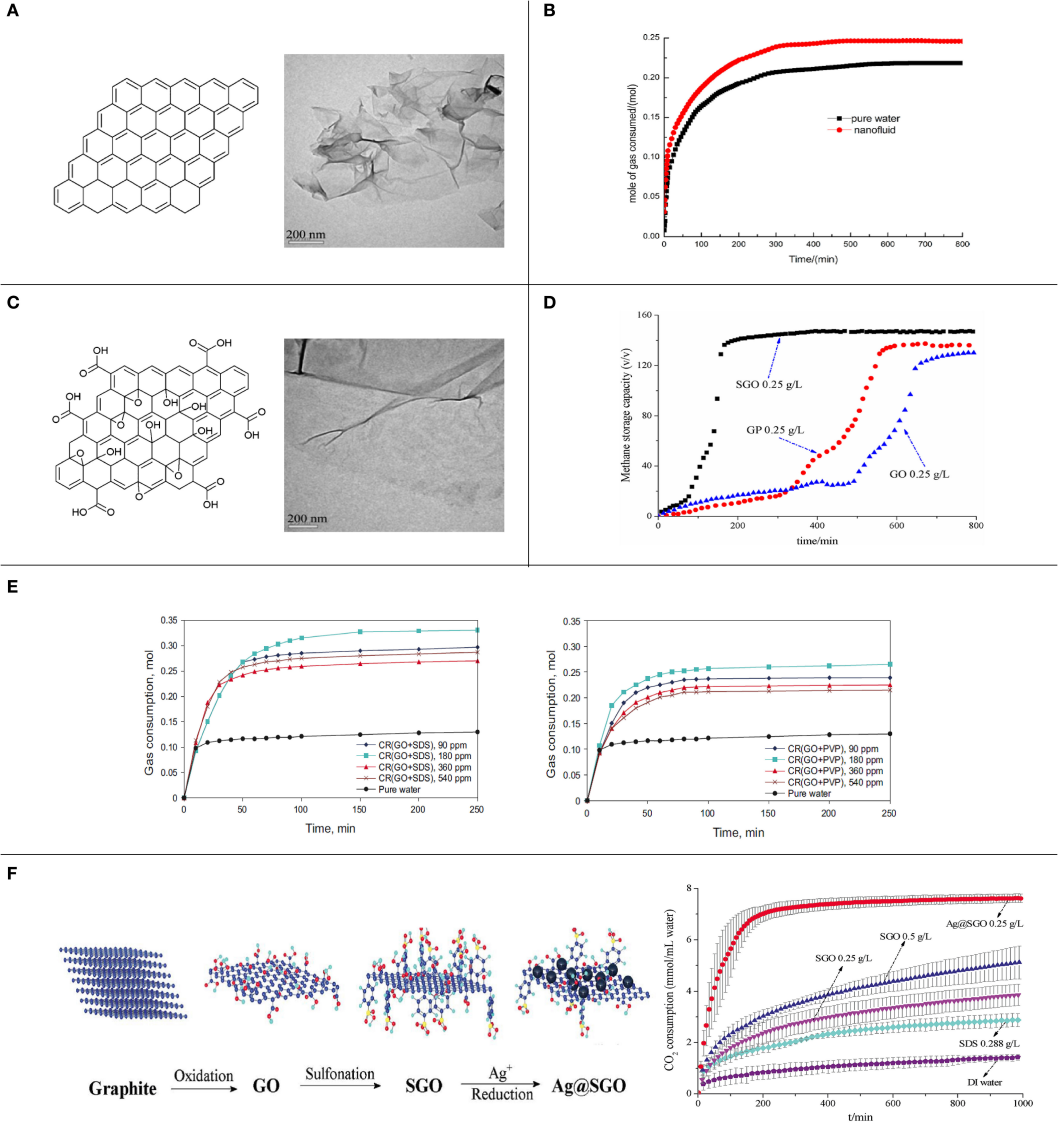
**(A)** Structural diagram and TEM image of graphene; reproduced from Wang et al. (2017) with permission from the American Chemical Society. **(B)** Gas consumption during CO_2_ hydrate formation with pure water and graphene nanofluid; reproduced from Zhou et al. (2014) with permission from the American Chemical Society. **(C)** Structural diagram and TEM image of GO; reproduced from Wang et al. (2017) with permission from the American Chemical Society. **(D)** Storage capacity during methane hydrate formation in the presence of graphene, GO, and SGO; reproduced from Wang et al. (2017) with permission from the American Chemical Society. **(E)** Gas consumption during methane hydrate formation at different concentrations of chemically RGO with SDS and PVP; reproduced from Abedi-Farizhendi et al. (2019b) with permission from the China University of Petroleum Beijing. **(F)** Structural diagram of graphite, GO, SGO, and Ag@SGO, and gas consumption during CO_2_ hydrate formation with different promoters; reproduced from He and Wang (2018), which was previously conducted by us and originally published by the Royal Society of Chemistry.

Graphene is a two-dimensional, single-layer nanosheet consisting of *sp*^2^ hybridized and honeycomb-arranged carbon atoms (Figure 2A; Huang et al., 2011). The peculiar layer structure and chemical structure endow graphene with remarkable properties, including large specific surface area, high transparency, excellent mechanical strength, and superior electrical and thermal conductivities, which enable graphene to permit a wide range of applications (Park and Ruoff, 2009).

Pristine graphene is highly hydrophobic and is impossible to directly disperse in water without assistance or dispersing agents, which constrains large-scale solution-based production and application processes (Li et al., 2008). Graphene derivatives, such as graphene oxide (GO) and chemically modified graphene (CMG), have been prepared. Compared to pristine graphene, graphene derivatives keep more oxygen-containing groups or other functional groups, which cause graphene derivatives to exhibit more appreciable dispersity and chemical reactivity (Huang et al., 2011). Graphene and its derivatives have been further incorporated into different functional materials to form graphene-based composites, which could be applied in the fabrication of field-effect transistors, sensors, clean energy devices, transparent conductive films, photocatalysis, etc. (Kumar et al., 2019). Graphene, graphene derivatives, and graphene-based composites are collectively known as graphene-based materials, all of which have admirable thermal and electrical properties as well as presenting a nanostructure and a large specific surface area (Figure 2).

## Graphene-Based Promotors of Gas Hydrate Formation

Due to their excellent properties, graphene-based materials might be exceptional promoters of gas hydrate formation: they can efficiently improve heat transfer by removing the heat generated during HFP and can meanwhile increase mass transfer due to their nanostructure and also accelerate nucleation by increasing inhomogeneity of the system, which consequently promote gas hydrate formation. The studies employing graphene-based materials as promoters of gas hydrate formation are shown in Table S1.

### Graphene

The hydrophobic graphene has been dispersed in water to prepare graphene nanofluid, and this has been used as a promoter of gas hydrate formation. Ghozatloo et al. (2015) studied the effects of graphene in natural gas hydrate formation and utilized 1 wt% of graphene nanofluid at initial conditions of 6.89 MPa and 277.15 K. The results showed that the graphene nanofluid could reduce the induction time by 61.07% and increase the storage capacity by 12.9% compared with pure water (Figure 2B). These enhancements might be attributable to the increase in initial dissolved gas in nanofluid, heterogeneous nucleation, and the heat transfer coefficient. Wang et al. (2017) used graphene nanofluid to promote methane hydrate formation at initial conditions of 6 MPa and 277.15 K with 300 rpm stirring and found that graphene (0.25–0.75 g L^−1^) reduced the hydrate formation period by 45–80% and improved the hydrate formation rate and the storage capacity by 190–660% and 45–70%, respectively, compared with pure water. The results implied that the graphene nanosheets not only increased heterogeneous nucleation in the system and provided abundant active sites for hydrate nucleation but also produced a high transfer efficiency that could remove the heat generated by hydrate formation from the system, consequently improving the efficiency of methane hydrate formation. Due to similar promotion mechanisms, graphite nanoparticles also have positive effects on gas hydrate formation. Zhou et al. (2014) applied graphite nanoparticles to promote CO_2_ hydrate formation at initial conditions of 3.5 MPa and 277.15 K with 300 rpm stirring and suggested that the induction time was decreased by 80.8% and the maximum CO_2_ consumption was increased by 12.8% in comparison to pure water. They argued that the high heat transfer coefficient and the large specific surface area of graphite nanoparticles played critical roles in promoting CO_2_ hydrate formation.

### Graphene Oxide

GO sheet is exfoliated from graphite oxide and has abundant oxygen-containing groups on its surface. GO also retains a single-layer structure, good thermal properties, and a huge specific surface area (Figure 2C). Compared to graphene, the thermal conductivity of GO is weakly decreased because of the existence of oxygen-containing groups that destroy the conjugated structure of nanosheets (Wang et al., 2017). However, GO is amphipathic and can act as a surfactant and presents superior dispersion in water; therefore, GO might be suitable for promoting gas hydrate formation (Yan et al., 2018).

GO has been immediately added into the system during HFP and functioned well in promoting gas hydrate formation. Rezaei et al. (2016) conducted ethylene hydrate formation with GO and SDS as promoters, revealing that GO was more effective in decreasing the induction time while SDS performed better in increasing the storage capacity. The minimum induction time, reduced by 96% compared to pure water, was obtained by 150 ppm of GO. The storage capacity failed to be promoted at a low concentration of GO (50 ppm), whereas it was markedly promoted at high concentrations of GO (150 and 150 ppm). Rezaei et al. argued that GO could provide an excellent structure for heterogeneous nucleation and a network pattern for the assembly of water and ethylene molecules. Additionally, its high specific surface area could improve mass transfer, and, meanwhile, the carboxyl and hydroxyl groups on GO could incorporate hydrogen bonds that further stabilize the hydrate crystals. Abedi-Farizhendi et al. (2019a) carried out propane hydrate formation in the presence of reduced graphene oxide (RGO) and GO, and the results indicated that they both improved the propane hydrate nucleation and formation rate while having no significant effects on storage capacity and water to hydrate conversion. The improvements could be attributed to the numerous nucleation sites, high specific surface area, and increased mass transfer produced by carbon nanostructures. Yan et al. (2018) adopted GO as a promoter of CO_2_ hydrate formation at 279 K and different initial pressures ranging from 3 to 5 MPa. They found that GO could shorten the induction time by 53–74.3% and increase the gas consumption by 5.1–15.9%. These effects were ascribed to the high heat and mass transfer efficiencies, high gas dissolution, and improved nucleation and formation rate.

However, a few studies found that GO also had inhibiting effects on gas hydrate formation. Kim et al. (2014) mentioned that the confinement and strong interaction of water caused by nano-sized pores and hydrophilic groups of GO could reduce water activity and therefore that the phase behavior of methane hydrates would be significantly inhibited. In another study, Wang et al. (2017) investigated the effects of GO on methane hydrate formation and revealed that GO exhibited poorer promotion effects on hydrate formation compared with graphene, which might possibly be attributable to the lowered thermal conductivity of GO and the reduced water activity caused by GO (Figure 2D).

Collectively, GO has favorable thermal conductivity, a nanostructure, and a large specific surface area, which could increase heat and mass transfers during HFP. Meanwhile, the stronger hydrophilicity of GO could accelerate its dispersion in water, which is convenient for the use of GO in an aqueous system during HFP. However, the hydrophilicity of GO also has inhibitory effects on HPF, as it reduces the water activity in the system and possibly inhibits gas hydrate formation. Fortunately, with abundant functional groups, GO has admirable dispersity and chemical reactivity and accordingly can be readily modified by chemical modification methods of its carbon backbone to produce CMG, which offers infinite possibilities for improvement of the properties of GO to make it more applicable for the promotion of gas hydrate formation.

### Surfactant-Stabilized Graphene

In addition to the direct promotion of gas hydrate formation, graphene can also be mixed with surfactants to generate surfactant-stabilized nanofluids. Graphene usually exhibits poor dispersity and stability during gas hydrate formation, causing weak performance and cyclability during promotion. Therefore, a mixture of graphene and surfactants has been employed, where the surfactants function as both stabilizer and co-promoter. Hosseini et al. (2015) used SDS to stabilize graphene nanofluid (1 wt%) to prepare an SDS/graphene promoter for natural gas hydrate formation and indicated that the SDS/graphene promoter reduced the induction time by 19.2% and increased the storage capacity by 7.6% compared to the SDS/water system. The reduction in induction time was attributed to the presence of heterogeneous nucleation and a high heat transfer coefficient, and the enhancement in storage capacity was considered to be due to the increased gas dissolution and heterogeneous active sites. Moreover, the addition of SDS could improve the stability of nanosheets in aqueous suspensions. Abedi-Farizhendi et al. (2019b) synthesized RGO with SDS and polyvinylpyrrolidone (PVP), respectively, which were applied to promote methane hydrate formation at initial conditions of 4.5 MPa and 273.15 K. The results showed that the synthesized promoters both significantly decreased the induction time and considerably increased the water to hydrate conversion while not changing the storage capacity (Figure 2E). On the one hand, the RGO might produce heterogeneous nucleation, which has a lower effective surface energy, causing lower free energy and a lower nucleation barrier than homogeneous nucleation, and is consequently more kinetically favorable than homogeneous nucleation. Additionally, the carbon nanostructures provide numerous nucleation sites to facilitate nucleation. On the other hand, the movement of carbon nanostructures decreased resistance in the gas–liquid interface. Therefore, the mass transfer was increased, leading to a reduced induction time. Yu et al. (2018) mixed graphite nanoparticles (GN, 0.4 wt%) with different concentrations of sodium dodecyl benzene sulfonate (SDBS) to prepare promoters and subsequently investigated the synergistic effects of GN and SDBS on the kinetics of CO_2_ hydrate formation. The experimental results showed that the gas consumption, hydrate storage, hydrate formation rate, and water to hydrate conversion were increased by 86.4, 35.8, 85.1, and 20%, respectively, in the presence of GN+SDBS (0.04%) compared in a pure water system. Adding SDBS into GN nanofluid could inhibit GN aggregation and greatly reduce the surface tension of the solution, making gas molecules dissolve in water more easily, which favored CO_2_ hydrate formation.

### Graphene-Carried Promoters

Due to its firm and stable carbon backbone, graphene can also serve as a nanocarrier to fabricate novel promoters of gas hydrate formation. Wang et al. (2017) grafted –${\text{SO}}_{3}^{-}$ onto graphene nanosheets through sulfonation to form an SGO promoter, and SGO performed more efficiently than graphene nanofluid and GO in promoting methane hydrate formation. Methane hydrate formation finished within 200–300 min with 0.25–0.75 g L^−1^ of SGO, and the storage capacity reached 140–150 v/v (Figure 2D). On the one hand, the majority of oxygen-containing groups were reduced during preparation, which removed the inhibition of water activity. On the other hand, –${\text{SO}}_{3}^{-}$-coated nanosheets could provide a large interface for methane molecule adsorption and water molecule association and therefore led to a rapid hydrate formation rate. Furthermore, a novel promoter named Ag@SGO has been synthesized by He and Wang through grafting Ag nanoparticles onto SGO nanosheets, and this was subsequently used as a promoter for CO_2_ hydrate formation. Under 0.25 g L^−1^ of Ag@SGO, most of the CO_2_ hydrate formation finished within 200–250 min, and CO_2_ consumption reached 7.62 ± 0.16 mmol mL^−1^ water at 1000 min, which was almost 2.6 times that with SDS (Figure 2F; He and Wang, 2018). Ag nanoparticles could provide additional active sites for nucleation as well-removing heat from the system and accordingly further facilitated the CO_2_ hydrate formation.

Summarily, using graphene as a nanocarrier for various functional groups and nanoparticles is an effective, flexible, and feasible approach to preparing novel promoters for gas hydrate formation and is well-worth further study.

## Conclusion and Prospects

In this review, the existing studies on graphene-based promoters of gas hydrate formation have been summarized, the beneficial properties and advantages of graphene-based materials have been emphasized, and the promotion mechanisms of graphene, GO, surfactant-stabilized graphene, and graphene-carried promoters have been discussed and analyzed. Graphene-based materials with admirable properties are capable of promoting gas hydrate formation: the heat generated during HFP can be removed by graphene-based materials because of their high thermal conductivity, which increases heat transfer in the system and avoids the destruction of hydrate crystals by high temperature; secondly, graphene-based materials with a large specific surface area can increase mass transfer during HFP via providing abundant active sites for nucleation; additionally, the appearance of graphene-based materials can increase inhomogeneity in the system, and the heterogeneous nucleation forms hydrate crystals more readily than homogenous nucleation, effectively promoting gas hydrate formation.

The existing studies on graphene-based promotion of gas hydrate formation were implemented in lab-scale experiments, so the promotion effects, stability, and cyclability of graphene-based promoters in gas hydrate formation need to be investigated in pilot tests, which could be conducted in future work. Additionally, further research can focus on grafting graphene/GO with functional groups to produce exceptional CMG or introducing functional nanoparticles (e.g., Ag and Fe_3_O_4_ nanoparticles) onto surfaces of graphene/GO, aiming to obtain novel graphene-based promoters with desirable properties to significantly promote gas hydrate formation. Moreover, because of its nanostructure and remarkable electrical conductivity, graphene might serve as a nano-sized electric rotor under an electric field that could effectively stir within nano-sized confinement spaces, which might possibly be applied for HFP promotion via improving mass transfer.

## Author Contributions

M-TS wrote and revised the manuscript. FW and G-DZ supervised and revised the manuscript. All authors contributed to the article and approved the submitted version.

## Conflict of Interest

The authors declare that the research was conducted in the absence of any commercial or financial relationships that could be construed as a potential conflict of interest.
