# Supplementary material for: Graphene-Based Kinetic Promotion of Gas Hydrate Formation
Source: Front Chem. 2020 Jun 19;8:481. doi: 10.3389/fchem.2020.00481 (PMC7317304; doi:10.3389/fchem.2020.00481)
Supplement: Supplementary file 1 [file Data_Sheet_1.docx]

**Supplementary Information**

**Graphene-based kinetic promotion of gas hydrate formation**

*Meng-Ting Sun ^1^, Guo-Dong Zhang ^1^, Fei Wang ^1*^*

*^1^ Shandong Engineering Laboratory for Preparation and Application of High-performance Carbon-materials, College of Electromechanical Engineering, Qingdao University of Science & Technology, Qingdao, China*

***Correspondence:***

*Fei Wang*

*elliot_wang@qust.edu.cn*

***First author:***

*Meng-Ting Sun*

[*sunmt@qust.edu.cn*](mailto:sunmt@qust.edu.cn)

**TABLE 1 |** List of studies employing graphene-based materials as promoters of gas hydrate formation

| Promotor | Dosage | P (MPa) | T (K) | Stirring (rpm) | Gas | Ref. |
| --- | --- | --- | --- | --- | --- | --- |
| Graphene nanofluid | 1 wt% | 6.89 | 277.15 | 0 | Natural gas | Ghozatloo et al. (2015) |
| Graphite nanoparticles | 0.4 wt% | 3.5 | 277.15 | 300 | CO_2_ | Zhou et al. (2014) |
| GO | 50, 150, 250 ppm | 1.4, 1.6 | 277.15 | 25 | Ethylene | Rezaei et al. (2016) |
| GO | 90, 180, 360, 540 ppm | 0.45 | 274.15 | 30 | Propane | Abedi-Farizhendi et al. (2019a) |
| GO | 20, 35, 50, 100, 200, 300 ppm | 3-5 | 279 | 400 | CO_2_ | Yan et al. (2018) |
| GO | 30, 180, 360, 540 ppm | 4.5 | 274.15 | 30 | CH_4_ | Abedi-Farizhendi et al. (2019b) |
| GO | 0.25, 0.5, 0.75 g L^-1^ | 6 | 275.15 | 300 | CH_4_ | Wang et al. (2017) |
| SDS/graphene | 1 wt% | 6.89 | 277.15 | 0 | Natural gas | Hosseini et al. (2015) |
| SDBS/Graphite | 0.4 wt% | 3.5 | 277.15 | 300 | CO_2_ | Yu et al. (2018) |
| SDS/RGO | 30, 180, 360, 540 ppm | 4.5 | 274.15 | 30 | CH_4_ | Abedi-Farizhendi et al. (2019b) |
| PVP/RGO | 30, 180, 360, 540 ppm | 4.5 | 274.15 | 30 | CH_4_ | Abedi-Farizhendi et al. (2019b) |
| SGO | 0.25, 0.5, 0.75 g L^-1^ | 6 | 275.15 | 300 | CH_4_ | Wang et al. (2017) |
| SGO | 0.25, 0.5 L^-1^ | 3.7 | 275.15 | 300 | CO_2_ | He & Wang (2018) |
| Ag@SGO | 0.125, 0.25, 0.5 L^-1^ | 3.7 | 275.15 | 300 | CO_2_ | He & Wang (2018) |
